# Changes in Muscle Oxygen Saturation Measured Using Wireless Near-Infrared Spectroscopy in Resistance Training: A Systematic Review

**DOI:** 10.3390/ijerph18084293

**Published:** 2021-04-18

**Authors:** Claudia Miranda-Fuentes, Luis Javier Chirosa-Ríos, Isabel María Guisado-Requena, Pedro Delgado-Floody, Daniel Jerez-Mayorga

**Affiliations:** 1 Department Physical Education and Sports, Faculty of Sport Sciences, University of Granada, 18011 Granada, Spain; cmiranda@unab.cl (C.M.-F.); lchirosa@ugr.es (L.J.C.-R.); 2 Faculty of Rehabilitation Sciences, Universidad Andres Bello, 7591538 Santiago, Chile; 3 Department of Nursing, Physiotherapy and Occupational Therapy, Faculty of Nursing, Group of Preventive Activities in the University Health Sciences Setting, University of Castilla-La Mancha (Universidad de Castilla-La Mancha/UCLM), 02071 Albacete, Spain; IsabelM.Guisado@uclm.es; 4 Department of Physical Education, Sports and Recreation, Universidad de La Frontera, 4811230 Temuco, Chile; pedro.delgado@ufrontera.cl

**Keywords:** resistance training, tissue saturation, hemoglobin

## Abstract

*Background:* This study aimed to report, through a systematic review of the literature, the baseline and final reference values obtained by near-infrared spectroscopy (NIRS) of muscle oxygen saturation (SmO_2_) during resistance training in healthy adults. *Methods:* Original research studies were searched from four databases (Scopus, PubMed, WOS, and SportDiscus). Subsequently, three independent reviewers screened the titles and abstracts, followed by full-text reviews to assess the studies’ eligibility. *Results:* Four studies met the inclusion criteria, data were extracted and methodological quality was assessed using the Downs and Black scale. Muscle oxygen saturation (% SmO2) during reported muscle strength exercises showed a decreasing trend after a muscle strength protocol; that is, before the protocol (range = 68.07–77.9%) and after (range = 9.50–46.09%). *Conclusions:* The trend of the SmO_2_ variables is to decrease after a muscle strength protocol. Studies are lacking that allow expanding the use of these devices during this type of training.

## 1. Introduction

Today, near-infrared spectroscopy (NIRS) has positioned itself in the field of physical activity and health as a valid, reliable and inexpensive wireless instrument [1,2,3,4,5,6,7]. In addition, this technology is capable of evaluating, in real time, the balance between muscle oxygen supply and its demand during physical exercise [8]. Technically, these devices illuminate the skeletal muscle with infrared light and detect the light reflected through it as a consequence of the amount of light absorbed by the tissue, recognizing variables such as oxyhemoglobin (O_2_Hb) and deoxyhemoglobin (HHb), as well as other derivatives such as total hemoglobin (tHb = O_2_Hb + HHb) and muscle oxygen saturation expressed in percentage (%) (SmO_2_ = [O_2_Hb]/[tHb] × 100), among others [9].

Studies using wireless NIRS date from around 2006 (Portamon; Artinis Medical System, Einsteinweg, The Netherlands) and since then, these instruments have been used in various sports registries to examine oxygen behavior during physical exercise [10]; despite this, it is only a few years ago that this emerging technology has been considered more frequently in the evaluation of muscle strength training. Currently, there is a small number of portable NIRS that are used in the sports market; among the most used, we find Portamon (Artinis Medical System, Einsteinweg, The Netherlands), Moxy Monitor (Fortiori Design, LLC, Hutchinson, MN, USA), BSX Insight (BSX Athletics, Austin, TX, USA) [10] and Humon Hex (Humon Beta, Dynometrics, Inc., Phoenix, AZ, USA).

There is a relationship between tHb and blood flow evaluated with Doppler ultrasound during exercise, observing a high association between changes in tHb and blood flow responses [11]. On the other hand, differences have been found in SmO_2_ according to the level of training, recognizing that this variable can be improved with specific physical exercise [12]. In particular, the use of NIRS in studies related to strength training has shown that when the number of repetitions performed with submaximal loads is increased, there is a restriction in blood flow to the effector’s muscle with a consequent lack in the oxygen supply, influenced by load and type of training [13,14,15]. On the other hand, it has been found that the behavior of SmO_2_ during muscle strength exercise can differ from one muscle to another depending on the type of fiber that conforms it; for example, the vastus lateralis (VL), the preferred muscle at the time of performing studies of muscle strength of the lower limb, has abundant type I fibers and, compared to the rectus femoris (RF) in an exercise performed until muscle failure, SmO_2_ decreased more in the VL than in the RF [16].

Today, resistance training is widely used in physical exercise and rehabilitation environments for its proven benefits in reducing injury rates, improving skills and performance in specific sports [17]; in addition to that, this training modality has proven to be a powerful tool to restore health conditions, which is why the World Health Organization (WHO) has including resistance training as part of its recommendation of physical exercise for the general population, due to the evidence demonstrated in the prevention of non-communicable diseases and others [18,19]. However, to our knowledge, no information has yet been found that clearly indicates the baseline data provided by NIRS and its possible variation during muscle strength training in healthy adults, information that could improve our understanding of the mechanisms that influence physical performance and fatigue during this type of exercise, since blood flow can vary depending on the workload and metabolic demand [20], conditioning the response of this tissue to physical exercise [21]. We know that this information would be very useful both in the field of sport and in rehabilitation [22], since we frequently use systemic response variables to refer to muscle work [23], ignoring the baseline behavior specifically evaluated through NIRS equipment. Therefore, the objective of the present study was to report, through a systematic literature review, the initial and final SmO_2_ reference values obtained by NIRS during resistance training in healthy adults.

## 2. Materials and Methods

### 2.1. Registration of Systematic Review

A systematic review of the literature was performed following the checklist for the Preferred Reporting Items for Systematic Reviews and Meta-Analyses (PRISMA) [24]. The original protocol was registered with the International Prospective Register of Systematic Reviews (PROSPERO) in November 2020 (Registration number: Nº CRD42020221935). The protocol registration occurred before any of the searches were conducted.

### 2.2. Search Strategy

In November 2020, two independent researchers (C.M. and I.G.) searched the academic electronic databases Scopus, PubMed, WOS, and SportDiscus for titles and abstracts using the keywords MeSH and non-MeSH for muscle strength and near-infrared spectroscopy using Boolean operators (AND/OR) (Table 1). All search results were extracted and imported into a reference manager (Mendeley, version 1.19.4).

### 2.3. Eligibility Criteria

The following inclusion criteria were used to select articles for the systematic review: (1) only full articles published in English in peer-reviewed journals; (2) quantitative, observational and experimental studies were considered; (3) studies conducted in healthy adult subjects (over 18 years), both sexes, without distinction of race, recreationally trained or elite athletes with or without training programs; (4) as an intervention, only studies that used resistance training as a form of intervention were included; (5) muscle oxygen saturation was considered studies were monitored by NIRS that will record pre- and post-intervention data; (6) we view resistance training as a type of strength-building exercise program that requires the muscles of the body to exert force against some type of resistance, such as weights or weight machine. Resistance exercise is a combination of static and dynamic contractions that involve shortening and lengthening of skeletal muscles [25].

The search had no publication date restriction and all studies which were (1) published in a language other than English were excluded; (2) where full access to the text and conference presentations, theses and books were not contained; they were duplicates, editorials, review papers and expert opinions; (3) the primary and/or secondary authors of the articles did not respond to email requests to provide missing and required information.

### 2.4. Selection Criteria

**Figure 1:**
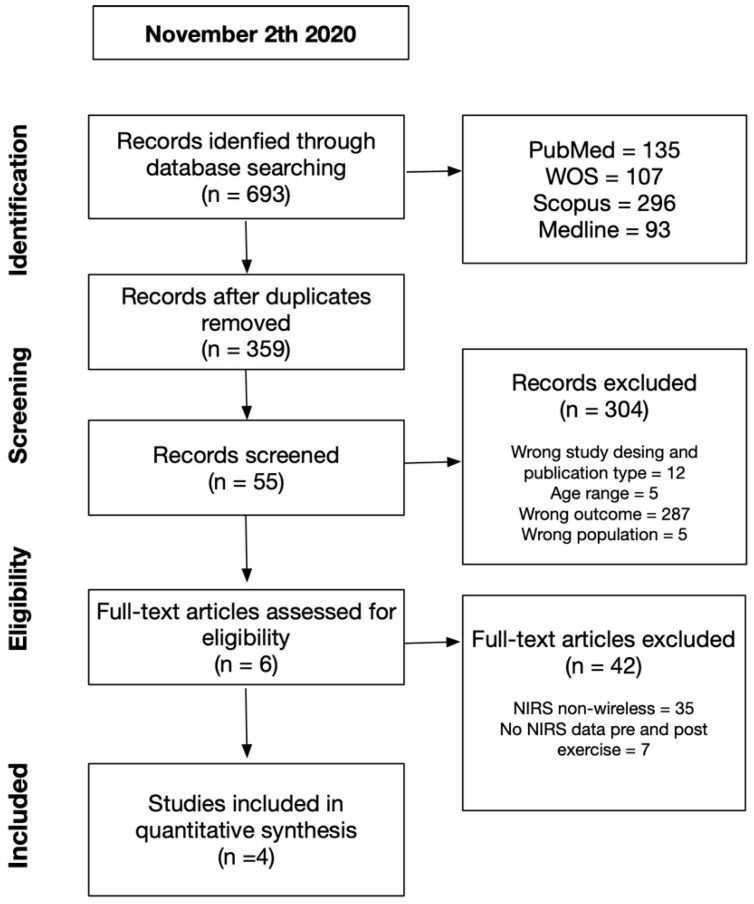
Summary of search strategy and selection process based on included and excluded studies.

After an initial screening for our inclusion and exclusion criteria and the removal of duplicates (C.M.), three reviewers independently assessed the articles by screening abstracts (C.M., I.G., D.J.). Next, the full text of each article was obtained and screened against the exclusion criteria. Any disagreement between them was resolved through discussion or the intervention of an arbitrary third investigator was requested (Figure 1). To maintain a correct order and communication in the review, the Rayyan^®^ application was used (available for free at http://rayyan.qcri.org; accessed on 1 December 2020). After this first stage, the previously selected articles were read in full. Subsequently, the finally selected articles were evaluated by two researchers (P.D. and D.J.) to review the evaluation of methodological quality using the Downs and Black scales [26] (Table 2).

### 2.5. Data Extraction

An Excel form was used for data extraction. Of each manuscript selected for review, the following information was considered: authors, date of publication, sample size, characteristics of the participants (age, sex, body mass, level of physical activity), type of evaluation of muscle strength, muscle(s), strength protocol, instrumentation and measured NIRS variables, a protocol for use of NIRS.

### 2.6. Procedures for Extracting Graphed Data

The data of some articles expressed in graphs were identified for review using the WebPlotDigitizer digitization program (https://automeris.io/WebPlotDigitizer/; accessed on 3 January 2021). Data extracted from digitizing software has been repeatedly shown to provide highly reliable and valid estimates from a single case investigation [27].

### 2.7. Assessment of Methodological Quality

Two reviewers (D.J. and P.D.) performed independent quality assessments using the modified Downs and Black [26]; in case of differences, a third researcher (C.M.) made the final consensus. The Downs and Black checklist is an index with high internal consistency and inter-rater reliability. A modified version of this scale was used because several items did not apply to the study design. Therefore, 15 of 26 items were chosen. For this review, studies were considered to be of high, medium and low quality if they met >75%, 60–75% and <60% of the applicable criteria, respectively [28] (Table 2).

**Table 2:** Quality assessment based on modified Downs and Black checklist.

Items from Modified Downs and Black Checklist																	
	Reporting							External Validity		Internal Validity						Total Score	Total as a %
Author	1	2	3	5	6	7	10	11	12	14	15	16	18	20	25		
Alvarez et al., 2020 [29]	1	1	1	1	1	1	1	0	0	0	0	1	1	1	0	10	63
Gómez-Carmona et al., 2020 [15]	1	1	1	0	1	1	1	0	0	0	0	0	1	1	0	8	50
Davis et al., 2020 [14]	1	1	1	0	1	1	0	0	0	0	0	0	1	1	0	7	44
Timon et al., 2020 [30]	1	1	1	1	1	1	0	0	0	0	0	0	1	1	0	8	50
**Total for each item**	4	4	4	2	4	4	2	0	0	0	0	1	4	4	0		
**%per each item**	100	100	100	25	100	100	25	0	0	0	0	13	10	100			

All questions were scored on the following scale: yes: 1, unable to determine: 0 and no: 0. For number 5: yes: 2, partially: 1, no: 0.

1. Is the hypothesis/aim/objective of the study clearly described?
2. Are the main outcomes to be measured clearly described in the Introduction or Methods section?
3. Are the characteristics of the patients included in the study clearly described?
4. Are the distributions of principal confounders in each group of subjects to be compared clearly described?
5. Are the main findings of the study clearly described?
6. Does the study provide estimates of the random variability in the data for the main outcomes?
7. Have actual probability values been reported for the main outcomes except where the probability value is less than 001 (i.e., indicating *p* = 0.043 rather than *p* < 0.05)
8. Were the subjects asked to participate in the study representative of the entire population from which they were recruited?
9. Were those subjects who were prepared to participate representative of the entire population from which they were recruited?
10. Was an attempt made to blind study subjects to the intervention they have received?
11. Was an attempt made to blind those measuring the main outcomes of the intervention?
12. If any of the results of the study were based on ‘data dredging’, was this made clear?
13. Were the statistical tests used to assess the main outcomes appropriate?
14. Were the main outcome measures used accurately (valid and reliable)?
15. Was there adequate adjustment for confounding in the analyses from which the main findings were drawn?

## 3. Results

### 3.1. Quality Assessment

Quality assessment scores ranged between 44% and 63% (7 to 10 out of 16 points) with a mean of 52% (Table 2). One article was rated as high quality and three articles were rated as low quality. All studies received points for items 1, 2, 3, 6, 7 (reporting) and 18, 20 (internal validity). On the other hand, none of the articles scored positively concerning items 11, 12 (external validity) and 14, 15, 25 (internal validity).

### 3.2. Included Studies and Study Characteristics

Finally, four articles met the inclusion criteria established for this review and, in total, reported the results of the work with NIRS during muscle strength exercises of 42 subjects with an age that ranged between the range of 21 and 23 years, where 85.71% corresponded to men and 14.28% to women. The subjects recruited by the studies were physically active, muscular strength trained, and healthy (Table 3).

Regarding muscle strength evaluation results, three of the four studies considered the evaluation of 1RM for the load prescription of their protocols and one study evaluated the maximum voluntary contraction (MVC). The four publications evaluated lower extremities through Squat-type exercise modalities, not exceeding 75% load regarding the type of protocol used.

Three of the four articles used MOXY brand NIRS equipment in different models, and one study used PortaMon; crosswise, all the articles delivered results in %, although each one separately also provided other additional units of measure; the vastus lateralis is the preferred site for positioning the NIRS devices in the four studies reviewed (Table 4).

Finally, SmO_2_ (%) data during reported muscle strength exercises varied before the protocol, between (range) 68.07–77.9%, and after training (range) 9.50–46.09%.

## 4. Discussion

This study aimed to report, through a systematic review of the literature, the baseline and final reference values obtained by near-infrared spectroscopy (NIRS) during resistance training in healthy adults. According with that, to our knowledge, this is the first systematic review to explore and summarize baseline and final baseline values obtained by NIRS during muscle strength exercises in healthy adults. As a result, SmO_2_ stands out as the most studied metabolic biomarker with NIRS devices, showing that this variable decreases as an acute response to muscular strength exercise, finding results before the strength protocol, between (range = 68.07–77.9%), and posterior (range = 9.50–46.09%). On the other hand, muscle oximetry in the evaluation of skeletal muscle performance during muscular strength exercises is an emerging area, as is supported by selecting the articles in this review and the years of their publication.

Regarding the diversity of devices used in the selected studies, the Moxy device was used in three of the reviewed articles [14,15,30] and one of them used Portamon [29]. One of the main characteristics of the Moxy and Portamon devices is that they can be attached manually to any muscle group using Velcro straps, which makes their use during a variety of activities such as cycling, running and, lately, training more functional muscle strength [4], unlike other devices such as The Wearable Lactate Threshold sensor (WLT) (BSXinsight multi-sport edition, Austin, TX, USA) which, from a functional aspect, must be used on the gastrocnemius muscle within a custom-made compression sleeve, which could limit its use [31]. Importantly, NIRS devices allow one aspect of the muscle response (SmO_2_) to be monitored during an exercise protocol. In the literature, the muscular response of SmO_2_ using NIRS during other physical exercise protocols has been highlighted, observing a significant correlation with the maximum lactate in steady state and the critical power [32] and the second ventilatory threshold [33], where the use of these devices has been indicated as a contribution in optimizing and managing the physical response of the athlete in endurance exercises [34,35]. However, despite the lack of research in the area of resistance training and SmO_2_, the compilation of information presented in this work may contribute to position NIRS devices as an instrument to be used during muscle strength training as a useful alternative, being functional and low cost during the evaluation and planning of muscle strength protocols, optimizing the prescription and management of physical performance in healthy adults.

Another relevant aspect to discuss is that in all the selected articles, SmO_2_ was evaluated in the vastus lateralis quadriceps, a coincidence that seems interesting to analyze when selecting a SmO_2_ evaluation protocol since the choice of muscles to evaluate this variable using a NIRS could influence the changes in muscle oxygenation and affect what is expressed by these devices, especially when large muscles with heterogeneous characteristics on their surface are evaluated; for example, literature has been found that evidence divergences for the VL versus rectus femoris quadriceps [36] or proximal versus distal gastrocnemius [37], therefore, special care must be taken regarding the use of a single site to predict the responses of the entire muscle or limb. In this same aspect, a review carried out by Perrey et al. [10] aimed to highlight the application of muscle oximetry in the evaluation of skeletal muscle performance; of the 57 studies selected by the researchers, 39 evaluated muscle oxygen saturation in the VL predominantly in aerobic sports and only 2 of them analyzed this variable in this same muscle during muscular strength training, appearing to be the preferred muscle in the NIRS assessment protocols, followed by deep flexor of the fingers, flexor carpus and gastrocnemius. Regarding this, Wang et al. studied the differences between the VL and the lateral gastrocnemius in the ability to assess the cut-off points of muscle oxygenation [38]; the difference between these two muscles may be due to anatomical and histochemical aspects, where the VL has fewer type I fibers and less citrate synthase (oxidative enzyme) activity than the lateral gastrocnemius; due to this, fast-twitch fibers would be recruited earlier than the VL when the workload increases continuously and incrementally, leading to increased anaerobic metabolism in the VL during moderate and high-intensity exercise [38]. Another difference between the VL and the lateral gastrocnemius could be the different patterns of use of the muscles during cycling, where the monoarticular muscles (VL) mainly participate in the generation of strength; while the biarticular muscles (lateral gastrocnemius) are responsible for the transmission of strength, the VL being more active than the lateral gastrocnemius in this sport and may be the reason for its preference [39]. However, these issues have not yet been fully resolved.

It is important to recognize that the interpretation of results provided by the different NIRS devices available on the market can be complicated by the diversity of this instrumentation regarding associated terminology (units of measurement and variables) and the general lack of protocol standardization, especially during muscle strength training, which to date, is a little-explored modality. However, the characteristics of the samples of the studies selected for this review are young adult subjects, physically active, without associated comorbidities and declaration of race; in the literature factors associated with these variables that can affect the signal of a NIRS finding, on the one hand, that in subjects with dark pigmentation skin (increased melanin concentration), this can reduce the signal emitted by the device [40]. On the other hand, the thickness of adipose tissue on skeletal muscle has also been shown to interfere with the results delivered by NIRS by reducing the relative contribution of the underlying skeletal muscle to the overall NIRS response, which may result in a greater intensity of the NIRS signal device due to reduced absorption by muscle chromophores [41]. Again, it seems to be a developing topic and future challenge for the next NIRS devices to hit the market.

Finally, another relevant aspect of the data collected refers to the decrease in SmO_2_ during muscle strength training, that is, before the protocol, between (range = 68.07–77.9%), and after exercise (range = 9.50–46.09%), which respond to the intensity of the applied muscle strength protocol, which does not exceed 75% of RM responding to a moderate load intensity. As reported in the literature, this result may be the cause of increased oxygen consumption by skeletal muscle during each protocol’s effort, which has been described, and can even increase up to 50 times during high-intensity training [42]. High-intensity exercise is characterized by metabolic profiles different from those of exercise performed at lower work rates; during strenuous exercise, decreases in intramuscular pH and phosphocreatine concentration, increases in blood lactate and intramuscular inorganic phosphate concentrations, and oxygen uptake (VO_2_) continue until muscle fatigue, reflecting an ineffective muscular metabolic system in the time [43]. It has been shown that during muscle strength exercise, increased intramuscular mechanical pressure can lead to reduced blood flow, which could result in transient muscle hypoxia [39], leading the subject to feelings of fatigue and decreased physical performance.

### Limitations and Strengths

Within the limitations of the present study, we highlight the limited literature selected according to the inclusion criteria proposed for this work, which do not allow us to conclude with greater detail and inference regarding the results found. In the literature, there is a lack of studies that focus on muscle oximetry during resistance training. Among the strengths of the study, we highlight that the use of wireless NIRS sensors in sports science can help to understand how skeletal muscle responds to stimuli from muscle strength training, providing new insights into baseline and posterior values in this type of intervention in healthy adults. In turn, we invite researchers to take the results obtained in this work and carry out future investigations that allow us to relate SmO_2_ with other biomarkers of metabolic stress during muscle strength training, in order to provide new knowledge and application of this variable for this type of exercise, following a similar path as other researchers have done [44,45,46,47,48], replicating these results in different populations and health conditions.

## 5. Conclusions

According to the inclusion and exclusion criteria, the variety in the methodology of the selected studies did not allow a large selection of articles and a greater degree of comparisons to present more detailed results. Although the included studies have similar probations, they used different NIRS devices and protocols; however, this review showed that the trend of SmO_2_ variables tends to decrease after a muscle strength protocol. Finally, although NIRS instrumentation has shown promise for assessing skeletal muscle performance when used in sports settings, there is still a need for further research development with randomized/longitudinal trials regarding the use of these instruments during strength training to support the already demonstrated advantages of the use of muscle oximetry in other sports and extrapolate them to protocols that include muscle strength exercises, enhancing its use as a complementary tool to monitor the response to this type of training with them to optimize the athlete’s performance.

## Figures and Tables

**Table 1:** Search terms and keywords utilized in each database search. Searches 1 and 2 were combined using ‘AND’.

Search 1	Search 2
“muscle strength” OR “resistance training” OR “strength training”.	“near infrared spectroscopy” OR “NIRS” OR “oximetry” OR “muscle oxygenation”.

**Table 3:** Characteristics of the participants of the studies selected for the review.

Author	Sample Size and Sex (Male/Female)	Age (Mean ± SD)	Weight (kg)	Height (m)	Level or Condition of Physical Activity or Health
Alvarez et al., 2020 [29]	12/NR	NA	NA	NA	Physically-active
Gómez-Carmona et al., 2020 [15]	12/NR	21.63 ± 1.17	77.76 ± 8.77	1.81 ± 0.08	Athlete
Davis et al., 2020 [14]	6/5	23.7 ± 1.4	NA	NA	Athlete
Timon et al., 2020 [30]	12/NR	21.1 ± 2.1	72.2 ± 7.2	1.77 ± 3.8	Healthy

NA *=* not available.

**Table 4:** Summary of findings from studies investigating muscle oxygen saturation before and after muscle strength exercise.

Authors	Measurement/Instrument to Assess Muscle Strength	Muscle Strength Protocol for NIRS	NIRS Device	Unit of Measurement	NIRS Protocol	SmO_2_ Pre or Min * (%)	SmO_2_ Post or Max * (%)	[tHb] Pre or Min *	[tHb] Post or Max *
Alvarez et al., 2020 [29]	Isokinetic dynamometer/(Humac Norm, CSMi Medical Solutions, Stoughton, MA, USA)	1 × 6 MVC at slow velocity (SV) (30 * s^−1^) and 1 × 6 MVC at fast velocity (FV) (180 * s^−1^), with 30 min rest between sets.	PortaMon	%; μM * s^−1^	Over VL (half the distance between the top of the patella and the femur’s trochanter).	68.07 ± 2.93 (SV); 66.76 ± 3.52 (FV)	46.09–45.53 ** (SV); 55.00–53.48 * (FV)	0.85 ± 1.71 (SV); 1.01 ± 1.52 (FV)	−10.93–11.15 ** (SV); 1.05–1.11 * (FV)
Gómez-Carmona et al., 2020 [15]	1RM Assessment Through Velocity-Based Estimation/A cable-extension linear velocity transducer (ChronoJump, Barcelona, Spain)	Squat exercise (4 sets of 4–16 rep at 60–75% 1RM and 40–80% of the level of effort	Moxy	%	Over VL (15 cm from the upper edge of the patella)	Range: 77.30–76.34	Range: 9.50–7.30	NA	NA
Davis et al., 2020 [14]	1 RM	3 sets of 15 rep at 70% of their 1-RM weight for both back (BS) and front squats (FS)	Moxy	%	Over VL, approximately 94 mm superior to the patella.	77.9–68.6 (BS), 83.3–67.9 (FS)	24.7–22.2 (BS), 24.7–22.2 (FS)	NA	NA
Timon et al., 2020 [30]	1RM was estimated from a 1–5 RM with Brzycki’s predictive equation.	Two protocols: 3 × 8 barbell squat (BS) with 3 min rest between each series; 3 × 8 bilateral maximum effort rep and 3 min rest between sets of flywheel YoYo^®^ squat (FS)	Moxy	%, g/dL	VL muscle belly, 12 cm above the lateral epicondyle of the right leg	73.6–75.4 (BS); 76.5–74.7 (FS)	19.9–20.4 (BS); 12.1–12.2 (FS)	(g/dL) 12.4–12.5 (BS) **; 12.5 (FS) **	(g/dL) 12.2–12.3 (BS) **; 12.1–12.2 (FS) **

Abbreviations: Data presented as mean ± standard deviation or range; MVC = maximum voluntary contraction; VL = vastus lateralis muscle; NA = not available; ** Evaluated with WebPlotDigitalizer. * min or pre = minimum or pre-exercise value. μM = Micromolar; % = percentage; g/dL = grams per deciliter.
